# Buprenorphine and cannabidiol co-administration reduces survival in a mouse model of orthopedic trauma

**DOI:** 10.3389/fphar.2025.1683842

**Published:** 2025-09-11

**Authors:** Caroline Bouchard, Geneviève Frégeau, Ian Massé, Louis De Beaumont

**Affiliations:** ^1^ Research Center, Hôpital du Sacré-Cœur de Montréal, Montreal, QC, Canada; ^2^ Department of Surgery, Université de Montréal, Montreal, QC, Canada

**Keywords:** orthopedic trauma, tibial fracture, buprenorphine, cannabidiol, survival rate

## Abstract

**Introduction:**

Analgesic selection following orthopedic trauma presents unique challenges due to potential drug interactions and physiological stress. The impact of different analgesic regimens - buprenorphine, cannabidiol (CBD), their combination, or vehicle - on survival was investigated in a murine model of tibial fracture.

**Methods:**

Eighty male C57BL/6 mice were randomly assigned to one of four group: (1) Buprenorphine (0.1 mg/kg, administered subcutaneously every 12 h for 3 days) plus cannabidiol (CBD, 100 mg/kg, administered intraperitoneally once daily for 7 days); (2) CBD only; (3) Buprenorphine + vehicle; or (4) Vehicle only. All animals also received carprofen (20 mg/kg, subcutaneously, once daily for 3 days). Survival was monitored over 7 days post-injury, and necropsies were performed to identify probable causes of death.

**Results:**

Following an orthopedic trauma, mice that received buprenorphine plus CBD exhibited significantly lower survival than those that received either treatment alone or vehicle only (p = 0.0049 and p = 0.02, respectively). No differences were noted between the other groups. Necropsy revealed gastrointestinal complications in most fatalities, while two deaths were linked to acute respiratory arrest post-injection.

**Discussion:**

These findings suggest that while buprenorphine and CBD are individually well-tolerated, their co-administration may increase the risk of adverse outcomes in murine orthopedic trauma models. Combining cannabinoids and opioids in translational research requires caution and emphasizes the need for mechanistic evaluation in preclinical models.

## 1 Introduction

Orthopedic traumas are among the most common causes of emergency room admissions worldwide, with the majority being lower limb injuries (Metsemakers et al., 2024). These traumas, including fractures, trigger inflammatory cascades, leading to severe outcomes in both clinical and experimental settings. Although inflammation is essential for tissue repair, excessive or prolonged responses can lead to complications. While standardized guidelines exist for managing acute pain postinjury and ensure prompt recovery, the optimal analgesic strategy in orthopedic trauma models remains an area of active investigation (Grzelak et al., 2022).

Non-steroidal anti-inflammatory drugs (NSAIDs) relieve pain by cyclooxygenases (COX) inhibition but may impair fracture healing by blocking prostaglandin-driven bone formation and resorption (Wheatley et al., 2019). Although patients still benefit from NSAIDs, clinicians are increasingly cautious with high doses, prolonged courses, or use in vulnerable groups (Ryan et al., 2024). Carprofen, a selective COX-2 inhibitor widely employed in animal research, likewise hinders bone formation when administered chronically (Huss et al., 2019).

Buprenorphine, a widely used analgesic in laboratory animal research due to its potent and long-lasting effects (Christoph et al., 2005), is a partial agonist at the μ-opioid receptor (MOR) and an antagonist at δ- and κ-opioid receptors. Beyond its use in preclinical and veterinary contexts, buprenorphine is also a cornerstone therapy in the treatment of opioid use disorder (Shulman et al., 2019). Its partial agonist activity at the MOR provides effective craving suppression and relapse prevention while reducing the risk of overdose compared to full opioid agonists (Gudin and Fudin, 2020). This dual relevance in both laboratory research and clinical practice underscores the importance of understanding buprenorphine’s pharmacological interactions, particularly when combined with other agents such as cannabinoids. However, its pharmacodynamic profile, particularly its suppressive effects on gastrointestinal (GI) motility, can complicate its use in orthopedic trauma models (Wolter et al., 2023). Moreover, opioids have been associated with negative effects on bone remodeling, including a decrease in bone mineral density (Coluzzi et al., 2015).

Cannabidiol (CBD), a non-psychoactive component of *Cannabis sativa*, has attracted interest for its anti-inflammatory, neuroprotective, and analgesic potential. Although it does not directly activate MOR, CBD is known to indirectly modulate opioid pathways (Arantes et al., 2024). Preclinical studies have shown that it exerts analgesic effects in neuropathic conditions by inhibiting the enzyme fatty acid amide hydrolase (FAAH) (Mlost et al., 2020; Peng et al., 2022), thereby increasing levels of anandamide, an endogenous cannabinoid neurotransmitter, and by activating the peroxisome proliferator-activated receptor gamma (PPARγ). CBD also reduces microglial activation in mild traumatic brain injury (mTBI) by decreasing dopamine uptake (Aychman et al., 2023), suppressing pro-inflammatory cytokine release, and mitigating neurotoxicity (Donat et al., 2017). CBD also exhibits antioxidant effects by modulating glutamate release, the brain’s primary excitatory neurotransmitter (Santiago-Castaneda et al., 2022). Growing evidence highlight the therapeutic potential of CBD. Improvements in cognitive function and behavioral outcomes have been observed following CBD administration in cases of mTBI, alongside reductions in seizure frequency in epilepsy (Silva et al., 2020; Friedman et al., 2021; Dong et al., 2024). Notably, a recent study using a murine model of tibial fracture showed that low doses of CBD can both alleviate pain and enhance bone formation (Khajuria et al., 2023). Importantly, CBD is also characterized by a favorable safety profile, with few serious adverse effects reported in both preclinical and clinical studies (Bergamaschi et al., 2011; Ewing et al., 2019). However, preclinical data also indicate that CBD may induce dose-dependent adverse effects, such as hepatotoxicity, mitochondrial dysfunction, and oxidative stress, particularly at very high doses (e.g., >200 mg/kg in rodents) (Ewing et al., 2019; Drummond-Main et al., 2023; Tallon and Child, 2023). These findings suggest the existence of a safety threshold beyond which the risk of toxicity increases. In contrast, clinical trials have consistently shown that CBD is well tolerated in humans at therapeutic doses ranging from 5 to 20 mg/kg/day, with only limited reports of serious adverse events (Devinsky et al., 2017; Devinsky et al., 2018a; Devinsky et al., 2018b). Together, these observations support the importance of continued translational research on CBD, provided that dosing regimens are carefully selected and adjusted for species-specific pharmacokinetic variations.

Recent investigations suggest that combining opioids and cannabinoids may provide synergistic analgesia, but the potential for adverse interactions remains poorly characterized, especially in complex injury models (Vierke et al., 2021).

In this Brief Research Report, the impact of four analgesic regimens - carprofen alone, or combined with buprenorphine, CBD, or both - was investigated on survival in a murine orthopedic trauma model of tibial fracture. Mortality rates and necropsy findings were evaluated to characterize potential cumulative toxicity and drug interaction effects. These findings may help inform best practices for analgesic selection in preclinical models of tibial fracture and highlight the importance of rigorous safety evaluation in translational research.

## 2 Methods

### 2.1 Ethical approval, animal housing, and husbandry

All procedures were approved by the local Animal Care Committee at Hôpital du Sacré-Cœur and conducted in accordance with the guidelines of the Canadian Council on Animal Care. Eighty male C57BL/6 mice (60–70 days old; Charles River Laboratories, Saint-Constant, QC, Canada) were housed individually in a temperature-controlled (22 °C–24 °C) and humidity-controlled (30%–40%) environment with a 12-h light/dark cycle. Mice had *ad libitum* access to standard rodent chow (Charles River, Rodent Chow 5075) and water, and were allowed to acclimate for at least 1 week prior to trauma induction.

### 2.2 Experimental groups and treatments

All CBD used in this study was Sundial CBD Isolate, obtained from the Société québécoise du cannabis. Multiple lots were independently analyzed for purity and concentration by the Plateforme de Bioanalyse de l’Institut de pharmacologie de l’Université de Sherbrooke to ensure consistency. CBD purity was confirmed to exceed 97.64% in all lots, as determined by liquid chromatography with diode-array detection. Detailed analytical reports are provided in the Supplementary Material S1, S2. Mice were randomly allocated to one of four treatment groups (n = 20 per group): (1) CBD + buprenorphine, intraperitoneal (i.p.) CBD (100 mg/kg once daily for 7 days) plus subcutaneous (s.c.) buprenorphine (0.1 mg/kg every 12 h for 3 days), (2) CBD only, i. p. CBD (100 mg/kg daily for 7 days), no opioid, (3) buprenorphine + vehicle, same buprenorphine schedule as above, plus daily i. p. injections of vehicle (1: 1: 18 ethanol: corn oil: saline), and (4) vehicle only, i. p. vehicle daily for 7 days. All animals received s. c. carprofen (20 mg/kg once daily for 3 days) as standard postoperative analgesia.

### 2.3 Tibial fracture and stabilization

Immediately prior to fracture induction, a local injection of a bupivacaine-lidocaine mixture (total dose: 1.5 mg/kg) was administered at the planned surgical site. Mice were positioned supine, and the right knee joint was exposed by gently separating adjacent muscles. The tibial plateau was punctured using a 25G needle, and a 0.45 mm stainless steel insect pin was inserted into the medullary canal to serve as an intramedullary stabilizer. A notch was then created in the tibial shaft using a #11 scalpel blade, and the fracture was completed using surgical scissors.

### 2.4 Health monitoring and endpoints

Recovery was assessed for 8 h post-injury using standardized criteria. Daily clinical assessments tracked weight, posture, coat condition, respiration, wound integrity, and nesting behavior (Supplementary Table S1). Predefined humane endpoints included ≥20% body weight loss, persistent ataxia, or total mortality rates exceeding 20%. Of note, one mouse had to be excluded in the vehicle-treated group due to an unstable fracture. All surviving animals were euthanized on Day 8 via intracardiac exsanguination under ketamine (120 mg/kg), xylazine (10 mg/kg), and isoflurane anesthesia.

### 2.5 Statistical analysis

Survival data were analyzed using Kaplan-Meier survival curves. Pairwise group comparisons were performed using the Mantel-Cox (log-rank) test in GraphPad Prism (version 10.2.3) to evaluate differences in mortality across analgesic regimens. A p-value <0.05 was considered statistically significant.

## 3 Results

### 3.1 Survival outcomes

**FIGURE 1:**
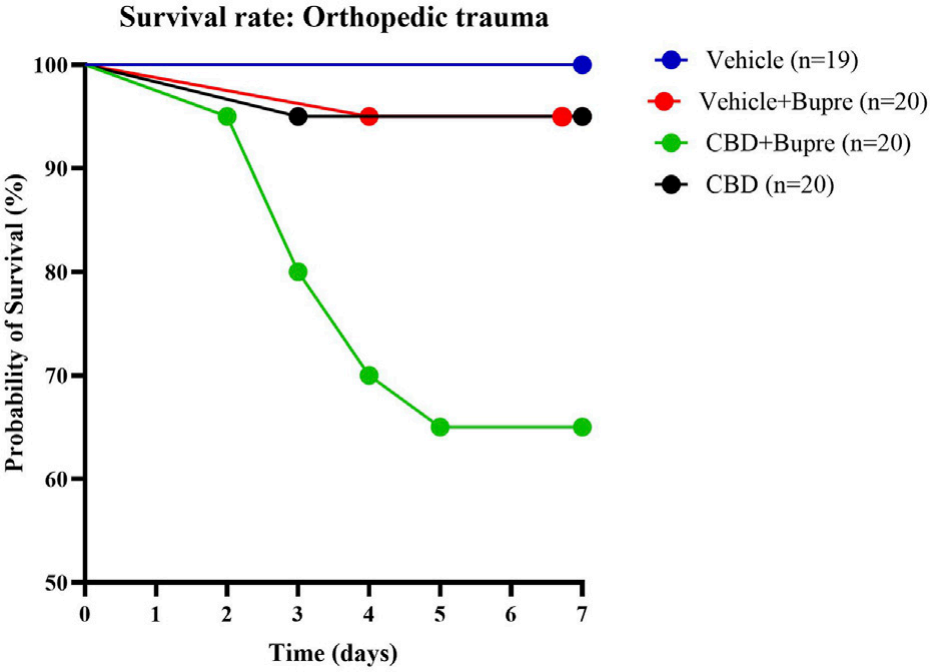
Mouse Survival Post-Surgery Survival curves of mice subjected to an orthopedic trauma. Mice were monitored for survival over 7 days post-surgery. Each curve represents a different analgesic regimen (CBD only, vehicle control + buprenorphine, combined CBD + buprenorphine or vehicle control only); n = 19-20 per group.

Survival was monitored daily for 7 days following orthopedic trauma induction. Mice were observed continuously for the first 8 h, then every 6 h during the first 24 h, and subsequently three times daily until the study endpoint. Day 7 survival rates were 65% for CBD + buprenorphine, 95% for CBD only, 95% for vehicle + buprenorphine and 100% for vehicle only (Figure 1). Pairwise comparisons revealed a statistically significant difference between CBD + buprenorphine and each of the other groups (p < 0.05), while no differences were observed among the other group comparisons. Comprehensive hazard ratios (HR) and confidence intervals (CI) for all pairwise comparisons are presented in Table 1.

**TABLE 1:** Pairwise comparisons of survival rates.

Comparison	Death HR	95% CI	p-value
CBD + Bupre vs. CBD	5.442	1.311–22.59	* 0.02
CBD + Bupre vs. VH + Bupre	5.609	1.354–23.22	* 0.0174
CBD + Bupre vs. VH	8.715	1.926–39.44	* 0.0049
CBD vs. VH + Bupre	0.026	0.064–16.41	0.9855
CBD vs. VH	7.029	0.1393–354.7	0.3297
VH + Bupre vs. VH	7.029	0.1393–354.7	0.3297

p-values, HR, and 95% CI, were calculated using the Mantel-Cox (log-rank) test. p < 0.05 was considered statistically significant. Abbreviations: Bupre, buprenorphine; VH, vehicle; HR, hazard ratio; CI, confidence interval.

### 3.2 Necropsy findings and causes of death

**FIGURE 2:**
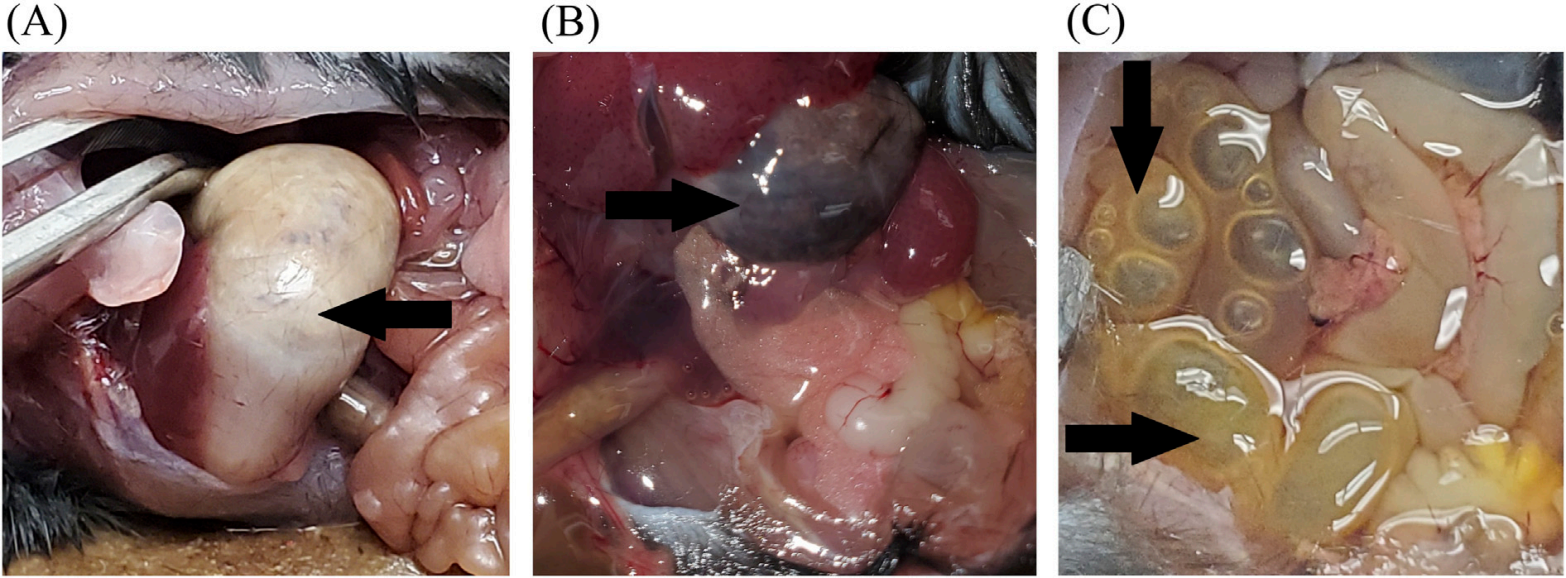
Representative Necropsy Findings Representative photographs of deceased mice showing necropsy findings. **(A)** Bloated stomach and empty intestines. **(B)** Discolored content in the stomach (black arrow). **(C)** Abdomen cavity showing full and distended intestines (black arrows). These observations were noted in several mice that died during the study and may suggest impaired gastrointestinal motility.

Of the nine total deaths, seven (78%) were attributed to gastric complications. When stratified by treatment group, GI complications occurred in 6 of 20 mice in the CBD + buprenorphine group and in 1 of 20 mice in the buprenorphine-only group. No GI complications were observed in the CBD-only (0/20) or the vehicle-only (0/19) groups. Five animals were found deceased, without any prior signs of distress. The remaining two, both in the CBD + buprenorphine group, exhibited serious health deterioration and were euthanized. Necropsy revealed marked stomach bloating (Figure 2A), discolored gastric contents (Figure 2B), and either empty or distended intestines with accumulated gas and fluid (Figure 2C). These findings are consistent with impaired GI motility, a known effect of MOR activation and possibly exacerbated by CBD’s influence on gut function. Respiratory complications were observed in the CBD + buprenorphine group (1 of 20 mice) and CBD-only group (1 of 20 mice). Both animals exhibited sudden collapse, postural loss, and labored breathing within 10 min of CBD administration, progressing rapidly to respiratory failure. Necropsy revealed no signs of tracheal obstruction, pulmonary edema, or hemorrhage, ruling out mechanical injury or aspiration as the cause.

## 4 Discussion

The present findings show that while buprenorphine and CBD are individually well tolerated in a mouse model of orthopedic trauma in the presence of carprofen, their co-administration reduces survival. This mortality pattern underscores the importance of investigating drug-drug interactions in preclinical models and translational research, especially with opioid-cannabinoid combinations.

### 4.1 Pharmacodynamic and pharmacokinetic considerations

The selection of analgesics in animal studies must be made carefully to minimize potential drug-drug interactions with other experimental compounds. Buprenorphine, a standard analgesic in laboratory research, is a partial agonist at the MOR and an antagonist at the δ- and κ-opioid receptors. MOR activation mediates its primary analgesic effect, while CBD modulates the endocannabinoid system indirectly, including through allosteric modulation and inhibition of FAAH. Therefore, co-administration of buprenorphine and CBD may increase the risk of adverse effects due to overlapping or interacting pharmacodynamic pathways. In addition, CBD has been reported to modulate immune and inflammatory signaling pathways, including microglial activation and cytokine release (Donat et al., 2017; Aychman et al., 2023), which may alter systemic responses to trauma and contribute to adverse outcomes.

The partial agonist nature of buprenorphine contributes to its variable analgesic potency and known ceiling effect on respiratory depression. This is particularly relevant in polytrauma models, where concurrent injuries can increase physiological stress and metabolic demand, potentially worsening adverse outcomes when multiple analgesics are combined. Additionally, buprenorphine and CBD share metabolic pathways, including glucuronidation via UDP-glucuronosyltransferase-2B7, and emerging clinical data suggest that cannabis use may elevate plasma concentrations of buprenorphine and its metabolites, likely through cytochrome P450 3A4 inhibition (Vierke et al., 2021).

Analgesic dosing regimens must be selected based on both translational relevance and an appropriate balance between efficacy and side effects. In this study, buprenorphine (0.1 mg/kg) was administered s. c. every 12 h for 72 h, while CBD (100 mg/kg) was administered i. p. once daily for 7 days. The 100 mg/kg i. p. dose of CBD was selected based on previous preclinical studies demonstrating both analgesic, anti-inflammatory and neuroprotective effects within this range (Kaplan et al., 2017; McCartney et al., 2020; Puighermanal et al., 2024). Using Food and Drug Administration -recommended Km factors, this dose corresponds to a human-equivalent dose of approximately 8.1 mg/kg (Reagan-Shaw et al., 2008), which falls within the therapeutic range (5–20 mg/kg) reported in clinical studies (Devinsky et al., 2017; Devinsky et al., 2018a; Millar et al., 2019). This dosage also accounts for the higher metabolic rate and shorter CBD half-life in rodents compared to humans (Dearborn et al., 2022). While this rationale supports the translational relevance of our dosing strategy, the absence of a dose-response analysis remains a limitation and restricts the generalizability of the findings.

Although some studies recommend administering buprenorphine every 4–8 h (Gades et al., 2000), others have demonstrated effective analgesia with longer intervals of 6–12 h (Schaap et al., 2012). The selected buprenorphine regimen (0.1 mg/kg s. c. every 12 h) represents a balance between ensuring adequate analgesia and minimizing handling, which can contribute to bone malunion in fracture models. A 12-h dosing interval was chosen as a pragmatic approach consistent with prior fracture studies. Nonetheless, it may have impacted systemic exposure and potential drug-drug interactions.

The route of administration also influences drug exposure and interaction potential. In this study, CBD was delivered i. p. and buprenorphine s. c., which differ in absorption kinetics and bioavailability. Further investigation is needed to determine how these differences may have influenced drug exposure and pharmacodynamic interactions.

Additionally, the prolonged administration of CBD could have led to cumulative toxicity, particularly in the presence of buprenorphine. Future studies should include pharmacokinetic profiling, liver enzyme measurements (e.g., alanine aminotransferase, aspartate aminotransferase), oxidative stress indicators, and systemic cytokine analysis to clarify the physiological burden of repeated CBD exposure.

Importantly, although CBD is a cannabinoid, its mechanism of action differs markedly from that of classical cannabinoid receptor 1 (CB1) receptor agonists. Unlike compounds such as Δ^9^-tetrahydrocannabinol or WIN 55,212-2, which directly activate CB1 receptors to exert central analgesic effects, CBD acts indirectly by inhibiting FAAH, increasing endogenous anandamide levels, and modulating other receptors like the transient receptor potential cation channel subfamily V member 1 (TRPV1), serotonin 1A (5-HT1A) receptor, and PPARγ. These mechanistic differences may explain variations in safety profiles and pharmacological interactions. Future studies should compare CBD with selective CB1 agonists to evaluate whether the adverse effects observed in this study are specific to CBD’s unique pharmacology or generalizable to cannabinoid-opioid combinations.

In summary, optimizing both survival and translational value in preclinical orthopedic trauma models requires a rigorous understanding of the pharmacodynamic and pharmacokinetic interactions between agents like buprenorphine and CBD. Analgesic selection must be informed by mechanistic insight, route of administration, dosing strategy, and cumulative exposure to minimize risk while maintaining efficacy.

### 4.2 Survival outcomes and mortality patterns

While survival was comparable between the buprenorphine-only, CBD-only and vehicle-only groups, it declined significantly when both drugs were co-administered. Across all animals, mortality remained low in the buprenorphine-only (1/20) and CBD-only (1/20) groups, but increased to 7/20 in the CBD + buprenorphine group. No death occurred in the vehicle-only group, where animals only received carprofen as standard postoperative analgesia. A significant difference was observed between the CBD + buprenorphine compared to each of the other groups (p < 0.05).

While this study does not assess the analgesic efficacy of the treatments, it focuses on identifying potential adverse effects that can lead to death when combining different therapeutic agents. Necropsy findings identified two primary causes of death: GI adverse effect (7 of 9 cases) and acute respiratory distress following injection (2 of 9 cases). Buprenorphine is known to reduce GI motility (Imam et al., 2018), albeit to a lesser extent than full MOR agonists such as morphine (Khanna and Pillarisetti, 2015), and is a recognized contributor to opioid-induced constipation (Imam et al., 2018). CBD has also been associated with altered gut motility, modulation of intestinal secretion, and microbiota dysregulation, likely via activation of TRPV1 and 5-HT1A receptor pathways (Crowley et al., 2024). Although NSAID are generally associated with GI complications, selective COX-2 inhibitors like carprofen have substantially lower risk compared to non-selective COX inhibitors (Dubois et al., 2004). The high rate of GI complications observed in the CBD + buprenorphine groups may therefore reflect cumulative adverse effects on gut function rather than a direct pharmacokinetic interaction alone. The high mortality rate in combined treatment groups suggests additive or synergistic effects on GI physiology, rather than isolated toxicity.

In contrast, the two deaths attributed to respiratory distress likely reflect opioid-induced respiratory depression (OIRD), a known adverse effect of MOR modulation (Dahan et al., 2018). Conditions such as orthopedic trauma can disrupt blood-brain barrier (BBB) integrity, potentially increasing central nervous system exposure to opioids and elevating the risk of OIRD (Varatharaj and Galea, 2017). Future studies should include arterial blood gas analysis and plethysmography to assess respiratory function more precisely. Additionally, histopathological evaluation of BBB tight junction proteins (e.g., claudin-5, ZO-1, occludin) could clarify whether barrier disruption correlates with survival outcomes.

To further characterize potential toxicity, histological analysis of the liver and GI tract is warranted to assess organ-specific damage from CBD-buprenorphine coadministration.

Although these findings suggest that co-administration of CBD and buprenorphine increases mortality risk, CBD may still confer benefits in tibial fracture models when administered without opioids or under alternative dosing strategies. Future studies should refine dosing protocols and evaluate both analgesic efficacy and safety to optimize translational relevance in preclinical orthopedic trauma models.

### 4.3 Implications for analgesic selection in complex injury models

Analgesic regimen selection has a critical impact on survival outcomes in preclinical models of orthopedic trauma. While the precise nature of the pharmacodynamic relationship between opioids and CBD, whether synergistic or antagonistic, remains to be fully elucidated, data suggest that their combination may pose heightened risk and should be approached with caution. Future studies should aim to clarify potential pharmacokinetic interactions that could influence systemic drug exposure and to optimize dosing strategies that minimize cumulative toxicity without compromising analgesic efficacy.

Pharmacokinetic profiling remains an important avenue for clarifying potential drug-drug interactions between CBD and buprenorphine. Future studies should incorporate plasma concentration measurements of CBD, its primary metabolites (7-hydroxy cannabidiol and 7-carboxy cannabidiol), buprenorphine and norbuprenorphine, at multiple time points to assess systemic exposure and metabolic clearance. These data would help determine whether CBD modifies buprenorphine pharmacokinetics in a manner that contributes to the increased mortality observed in this model.

Equally important is the integration of standardized nociceptive outcome measures to ensure that safety-focused protocols do not inadvertently reduce pain control. Given the increased physiological burden associated with overlapping injuries in polytrauma models, careful monitoring of GI function, respiratory parameters, and neurological recovery is essential. These considerations are key to advancing the development of safer, more effective multimodal analgesia protocols with enhanced translational relevance for complex clinical scenarios.

### 4.4 Study limitations

The universal administration of carprofen, while ethically necessary for postoperative analgesia, represents an important limitation. While it also reflects clinical practices, carprofen is known to influence GI physiology, and its concurrent use with buprenorphine or CBD may have contributed to the GI complications observed. This potential confounding effect limits the ability to attribute mortality exclusively to CBD-buprenorphine interactions. Future studies should therefore include groups without NSAID background treatment to better isolate drug-specific effects.

An additional limitation of this study is the possibility that neuropathic lesions resulting from the traumatic procedure may have contributed to the observed outcomes. Although this factor was not directly assessed, it cannot be excluded as a contributor to mortality or complications. Recognizing this possibility also highlights an avenue for future translational research into nerve repair mechanisms (Chiaramonte et al., 2023).

Another important limitation is the absence of detailed histopathological and biochemical analyses. Although gross necropsy suggested GI and respiratory complications, these findings cannot establish causality. Future studies should therefore include systematic histological evaluation of GI, hepatic, pulmonary, and central nervous system tissues, alongside biochemical markers such as liver enzyme activity, oxidative stress indices, and assessments of BBB integrity. Measures of systemic inflammatory burden may also provide mechanistical insights. Incorporating these approaches will be essential to define the causal pathways underlying the increased mortality observed with combined CBD-buprenorphine treatment.

Furthermore, the absence of validated nociceptive measures prevents direct comparison of analgesic efficacy across treatment groups. Future studies should incorporate established pain assays, such as von Frey testing or thermal withdrawal latency, to better evaluate the risk-benefit profile of each regimen.

Regarding the statistical results, it should be noted that while HR indicated significant group differences, several comparisons were accompanied by wide CI, reflecting instability of the estimates due to the relatively small sample size. This limitation reduces the robustness of the statistical claims and underscores the need for replication in larger cohorts to validate the observed mortality patterns.

## Data Availability

The original contributions presented in the study are included in the article/Supplementary Material, further inquiries can be directed to the corresponding author.
