# Supplementary material for: Buprenorphine and cannabidiol co-administration reduces survival in a mouse model of orthopedic trauma
Source: Front Pharmacol. 2025 Sep 11;16:1683842. doi: 10.3389/fphar.2025.1683842 (PMC12460340; doi:10.3389/fphar.2025.1683842)
Supplement: Supplementary file 1 [file DataSheet2.pdf]

Université de Sherbrooke
Institut de pharmacologie de Sherbrooke (IPS)

Plateforme de bioanalyse
Campus de la santé,
3001 12 Ave N, Sherbrooke,
QC J1H 5N4
https://www.usherbrooke.ca/ips/fr/plateformes/bioanalyse/

Plateforme de Bioanalyse

Rapport d'étude

**Nom du requérant :**	Louis De Beaumont
**À l'attention de :**	Daphnée Brazeau
**Projet :**	Profil de pureté
**Date du rapport :**	2022-10-24
**Version :**	1
**Nombre de pages :**	9

Si vous désirez de l'information concernant cette analyse, S.V.P. contacter : bioanalysis-fmss@usherbrooke.ca

***Notes**

- 2 composés de poudre de CBD ont été réceptionnés
- Profil de pureté déterminé en chromatographie liquide couplée à un détecteur à barrette de diode (LC-DAD)
- Profil de caractérisation déterminé en chromatographie liquide couplée à un détecteur de masse (LC-Qtof)
- Le rapport contient une annexe incluant les profils du blanc procédural et du matériau de référence (standards analytiques du CBD) analysé par LC-DAD et LC-Qtof

UNIVERSITÉ DE SHERBROOKE
Institut de pharmacologie de Sherbrooke (IPS)

Plateforme de bioanalyse
Campus de la santé,
3001 12 Ave N, Sherbrooke,
QC J1H 5N4
https://www.usherbrooke.ca/ips/fr/plateformes/bioanalyse/

Rapport d'analyse

1 Étude

Critères	Paramètres
Projet	Profil de pureté
Composé	CBD
Matrice	Poudre de CBD
Solvant de dissolution	Méthanol
Concentration	1 mg/mL
Pureté	Pureté déterminée par LC-DAD
Caractérisation	Caractérisation déterminée par LC-Qtof
Résultats	Profil d'impureté en UV et profil de caractérisation en HRMS

2 Résultats

2.1 Résultats obtenus pour l'échantillon 1 « Boite fermée » :

Référence échantillons 1 : « Boite fermée »	
Echantillons	Poudre de CBD
Structure	$C_{21}H_{30}O_2$
**Analyse pureté LC-DAD**	
Temps de rétention	8.60 min
% pureté obtenue	≥97.81 %
**Analyse caractérisation LC-Qtof**	
Electrospray	ESI +
Temps de rétention	8.60 min
m/z théorique	315.2318
m/z mesuré	315.2310
Δm (ppm)	2.5 ppm
Variation (ppm)	≤5.0 ppm

2.1.1 Profil d'impureté obtenu pour l'échantillon 1

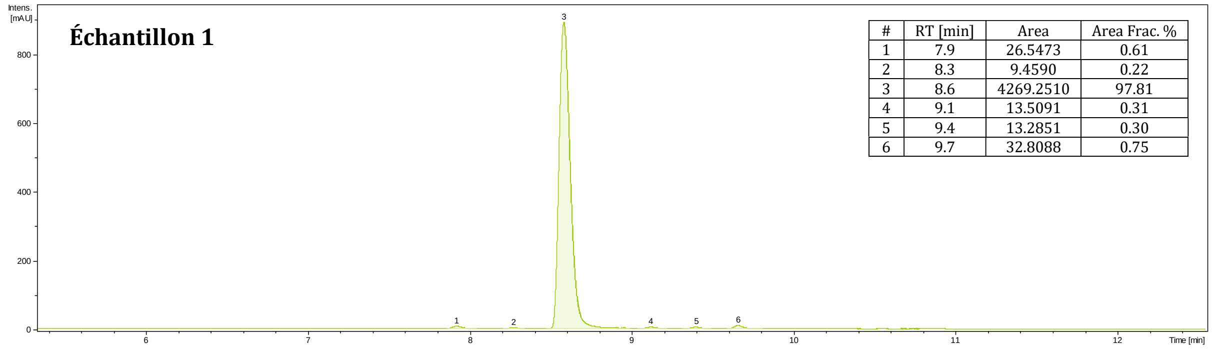

#	RT [min]	Area	Area Frac. %
1	7.9	26.5473	0.61
2	8.3	9.4590	0.22
3	8.6	4269.2510	97.81
4	9.1	13.5091	0.31
5	9.4	13.2851	0.30
6	9.7	32.8088	0.75

Figure 1: Profil de l'échantillon 1 obtenu en chromatographie liquide a barrette de diode (LC-DAD)

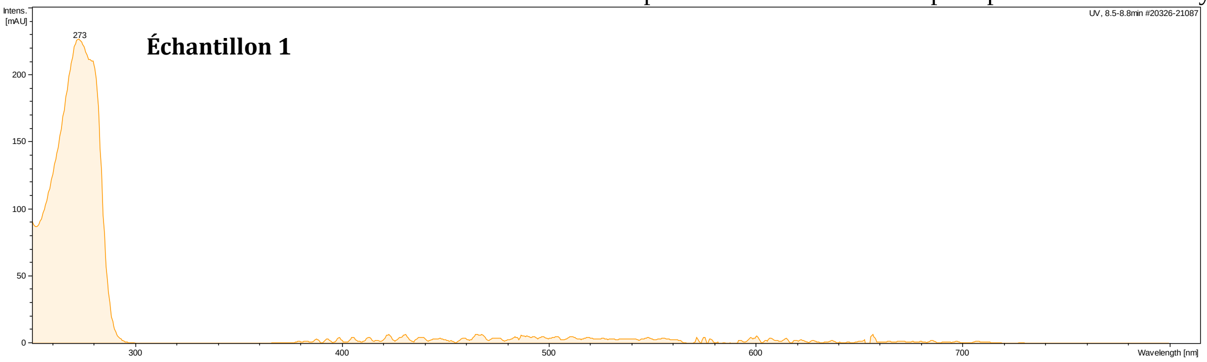

Figure 2: Spectre d'absorbance UV du composé CBD dans l'échantillon 1 majoritaire à 8.60 min

2.1.2 Profil chromatographique obtenu en HRMS pour l'échantillon 1

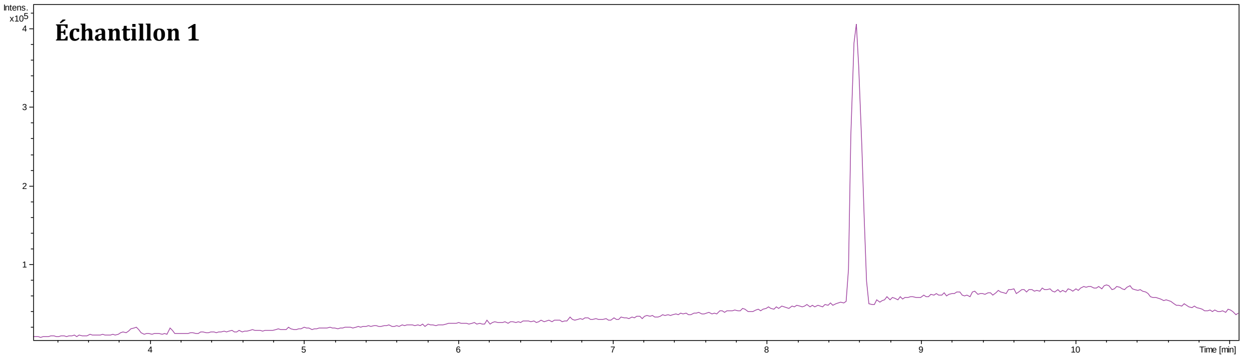

Figure 3: Profil chromatographique obtenu pour l'échantillon 1 en spectrométrie de masse à haute résolution (LC-Qtof)

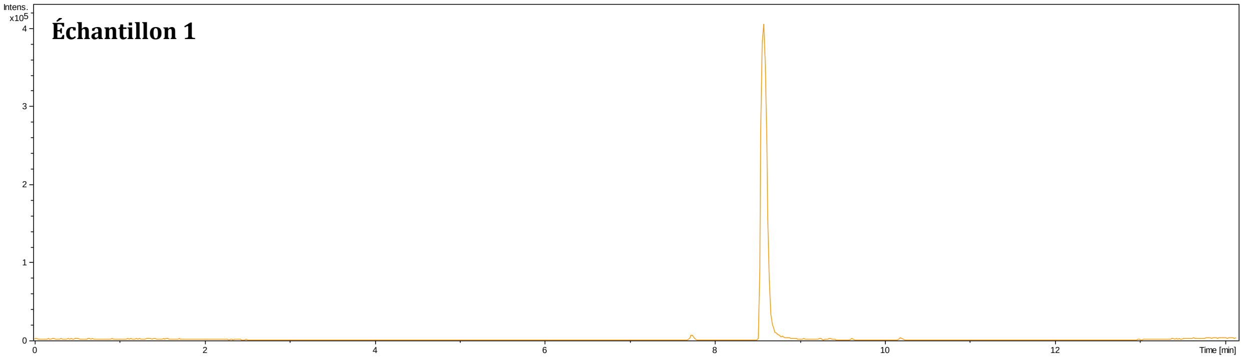

Figure 4: Masse extraite du composé majoritaire (m/z 315.2310) détecté à 8.60 min en spectrométrie de masse à haute résolution (LC-Qtof) présent dans l'échantillon 1

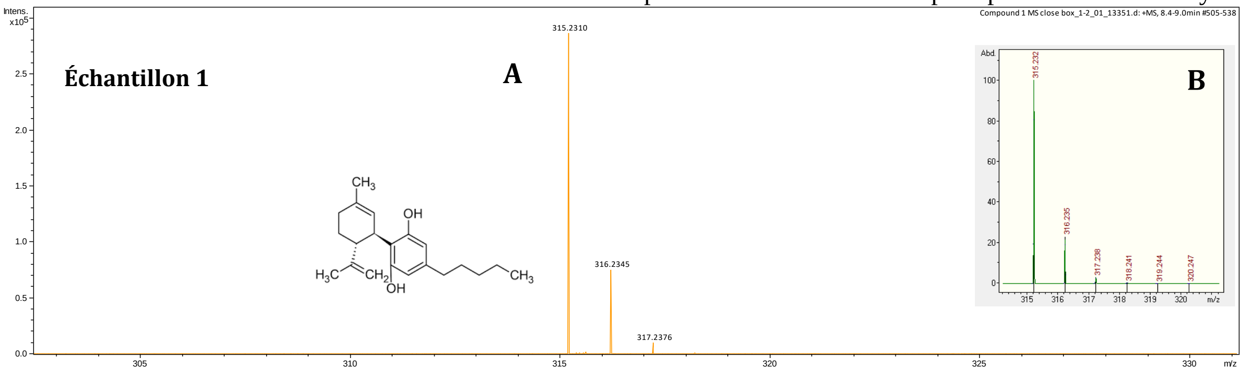

Figure 5: Confirmation du composé majoritaire CBD (m/z 315.2310) détecté à 8.60 min en spectrométrie de masse à haute résolution (LC-Qtof) avec le profil isotopique dans l'échantillon 1 **(A)**, profil isotopique théorique du composé CBD **(B)**

Un blanc procédural a été injecté et le profil en LC-DAD est présenté en annexe.

Un matériau de référence (standards analytique CBD) a été injecté afin de confirmer le profil UV en LC-DAD. Les profils sont présentés en annexe.

2.2 Résultats obtenus pour l'échantillon 2 « Boite ouverte » :

**Référence échantillons 2 : « Boite ouverte »**	
Echantillons	Poudre de CBD
Structure	$C_{21}H_{30}O_2$
**Analyse pureté LC-DAD**	
Temps de rétention	8.60 min
% pureté obtenue	≥97.77 %
**Analyse caractérisation LC-Qtof**	
Electrospray	ESI +
Temps de rétention	8.60 min
m/z théorique	315.2318
m/z mesuré	315.2317
Δm (ppm)	0.3 ppm
Variation (ppm)	≤5.0 ppm

2.2.1 Profil d'impureté obtenu pour l'échantillon 2

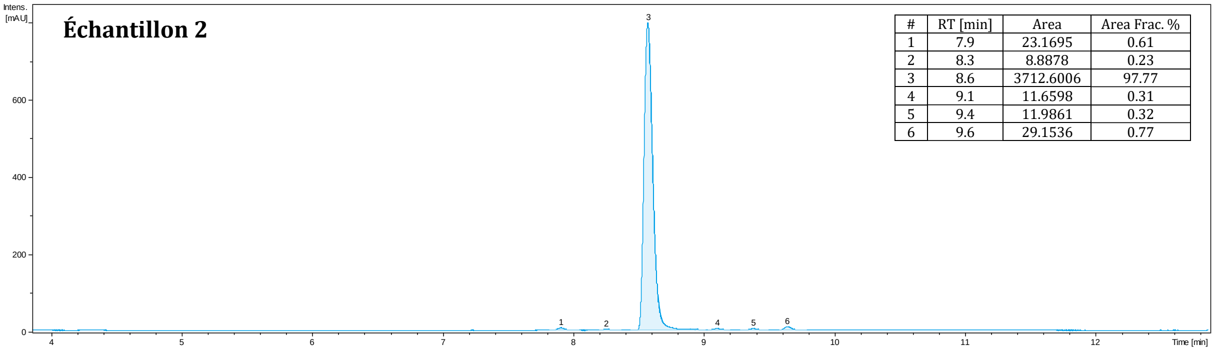

#	RT [min]	Area	Area Frac. %
1	7.9	23.1695	0.61
2	8.3	8.8878	0.23
3	8.6	3712.6006	97.77
4	9.1	11.6598	0.31
5	9.4	11.9861	0.32
6	9.6	29.1536	0.77

Figure 6: Profil de l'échantillon 2 obtenu en chromatographie liquide a barrette de diode (LC-DAD)

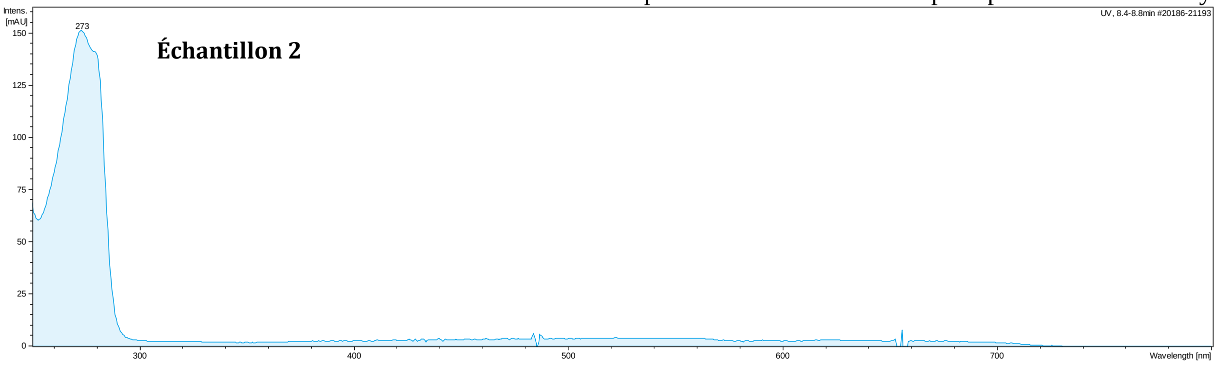

Figure 7: Spectre d'absorbance UV du composé CBD dans l'échantillon 2 majoritaire à 8.60 min

Un blanc procédural a été injecté et le profil en LC-DAD est présenté en annexe.

Un matériau de référence (standards analytique CBD) a été injecté afin de confirmer le profil UV en LC-DAD. Les profils sont présentés en annexe.

2.2.2 Profil chromatographique obtenu en HRMS pour l'échantillon 2

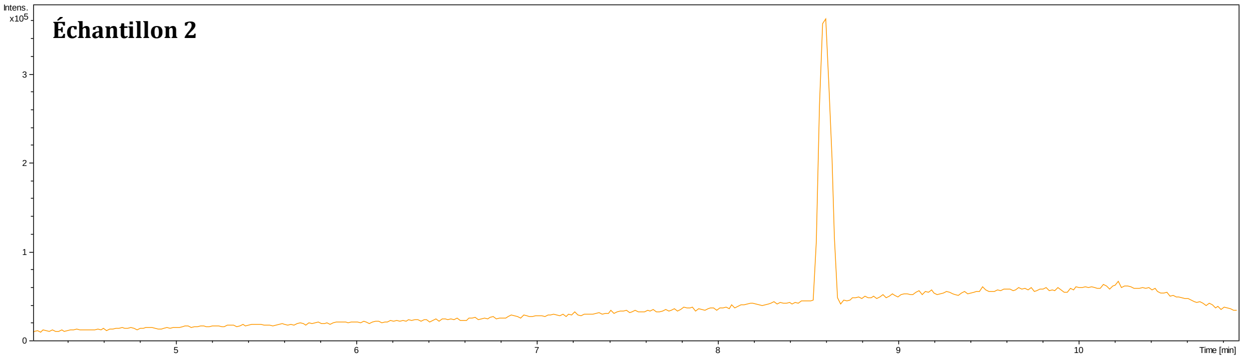

Figure 8: Profil obtenu pour l'échantillon 2 en spectrométrie de masse à haute résolution (LC-Qtof)

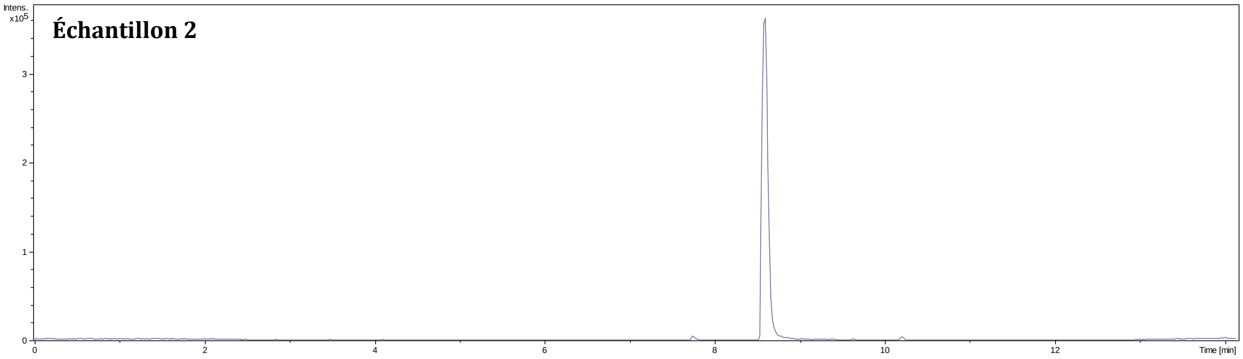

Figure 9: Masse extraite du composé majoritaire (m/z 315.2317) détecté à 8.60 min en spectrométrie de masse à haute résolution (LC-Qtof) présent dans l'échantillon 2

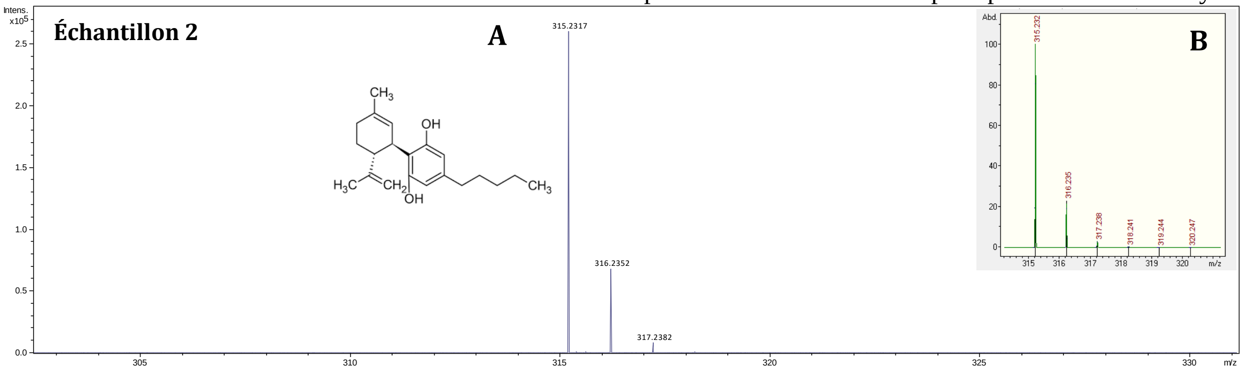

Figure 10: Confirmation du composé majoritaire CBD (m/z 315.2317) détecté à 8.60 min en spectrométrie de masse à haute résolution (LC-Qtof) avec le profil isotopique dans l'échantillon 2 **(A)**, profil isotopique théorique du composé CBD **(B)**

La caractérisation et la confirmation des échantillons de poudre de CBD ont été réalisée par LC-Qtof et les profils isotopiques des composés CBD sont comparés au profil isotopique théorique.

Un blanc procédural a été injecté et le profil en LC-Qtof est présenté en annexe.

Un matériau de référence (standards analytique CBD) a été injecté afin de confirmer la caractérisation et le profil de pureté d'un échantillon de CBD par LC-Qtof. Les profils sont représentés en annexe.

3 Bilan

- Le Pic majoritaire pour les échantillons 1 et 2 détecté à 8.60 min a été identifié et confirmé comme étant du CBD par spectrométrie de masse à haute résolution (LC-Qtof)
- L'incertitude sur la détermination de la masse exact est conforme aux critères d'acceptabilité (≤ 5 ppm)
- L'échantillons 1 de CBD montre une pureté ≥ 97.81 % en LC-DAD
- L'échantillons 2 de CBD montre une pureté ≥97.77 % en LC-DAD
- L'échantillon 1 et 2 montrent la présence de 5 impuretés présent à 7.9, 8.3, 9.1, 9.4 et 9.6 min.
- La présence d'impureté en LC-DAD n'ont pas pu être identifiées en LC-Qtof.
- Les données obtenues avec le matériau de référence en CBD sont conformes aux critères d'acceptabilité du fournisseur.

Plateforme de bioanalyse
Campus de la santé,
3001 12 Ave N, Sherbrooke,
QC J1H 5N4
https://www.usherbrooke.ca/ips/fr/plateformes/bioanalyse/

4 Annexe :

Cette section annexe regroupe les profils en LC-DAD et LC-Qtof du blanc procédural et du matériau de référence en CBD.

4.1 Profil du blanc procédural

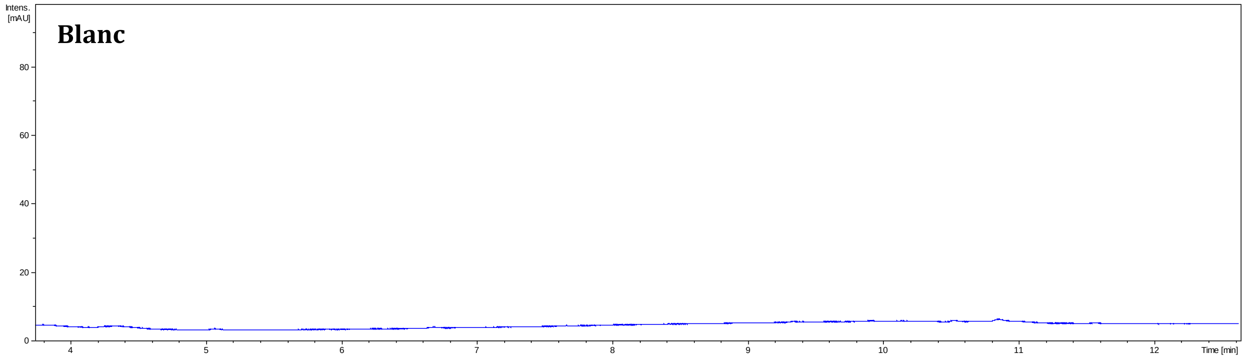

Figure 11: Profil du blanc procédural en chromatographie liquide a barrette de diode (LC-DAD)

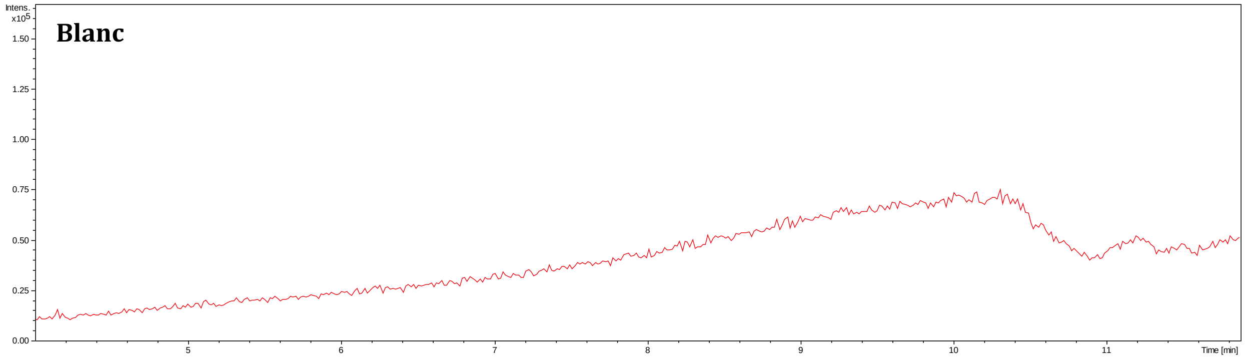

Figure 12: Profil du blanc procédural en spectrométrie de masse à haute résolution (LC-Qtof)

Institut de pharmacologie de Sherbrooke (IPS), Université de Sherbrooke
Plateforme de bioanalyse
Campus de la santé,
3001 12 Ave N, Sherbrooke,
QC J1H 5N4
https://www.usherbrooke.ca/ips/fr/plateformes/bioanalyse/

4.2 Profil du matériau de référence (standards analytique CBD)

Référence échantillons	Matériau de référence Standards analytique
Composé	CBD
Structure	$C_{21}H_{30}O_2$
Analyse	LC-DAD
Temps de rétention	8.60 min
% pureté théorique (certificat d'analyse du fournisseur)	99.7
% pureté obtenue	99.4
Δ	0.3%
Critère d'acceptabilité de la variation	≤0.5 %

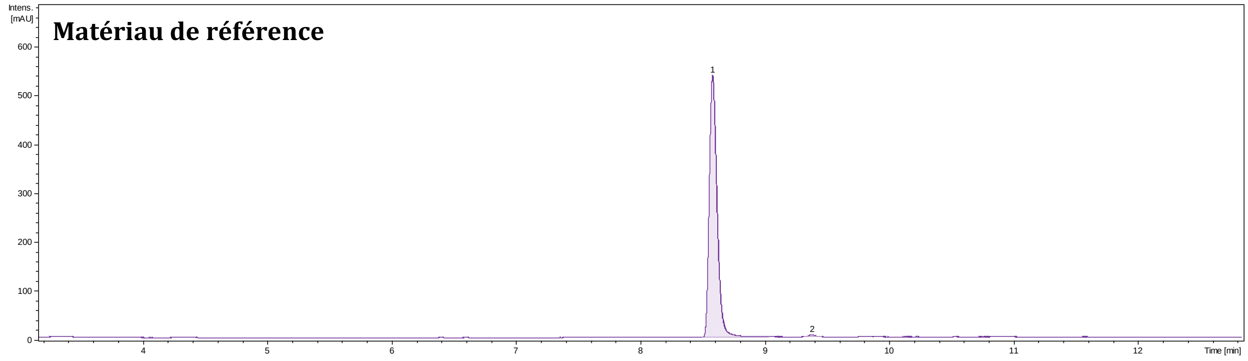

Figure 13: Profil du matériau de référence (standards analytique CBD) obtenu en chromatographie liquide a barrette de diode (LC-DAD)

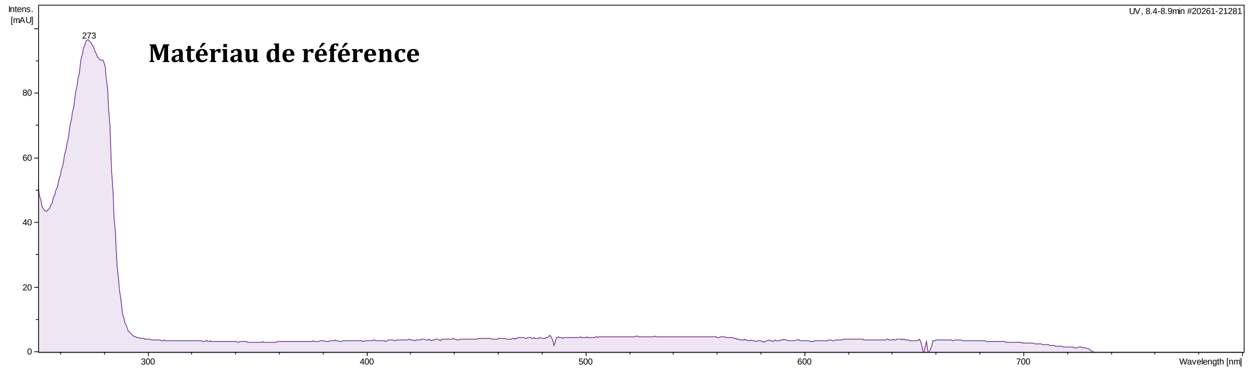

Figure 14: Spectre d'absorbance UV du matériau de référence (standards analytique CBD) à 8.60 min

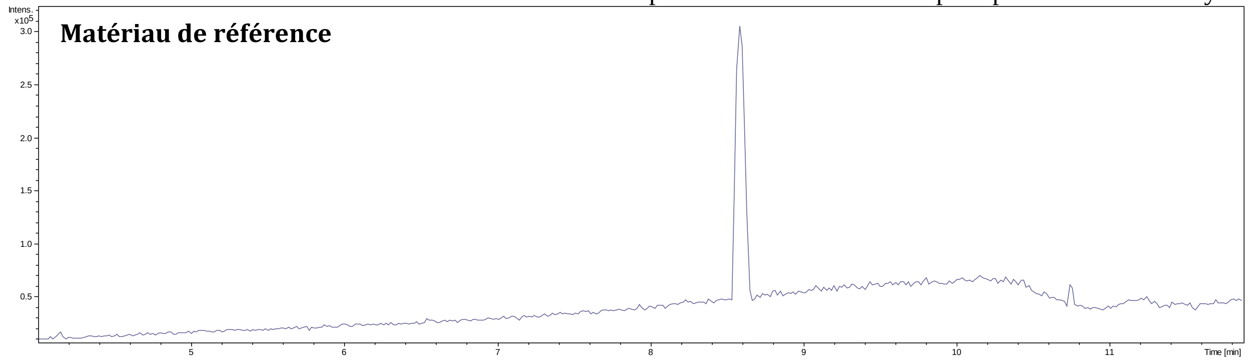

Figure 15: Profil chromatographique du matériau de référence (standards analytique CBD) obtenu en spectrométrie de masse à haute résolution (LC-Qtof)

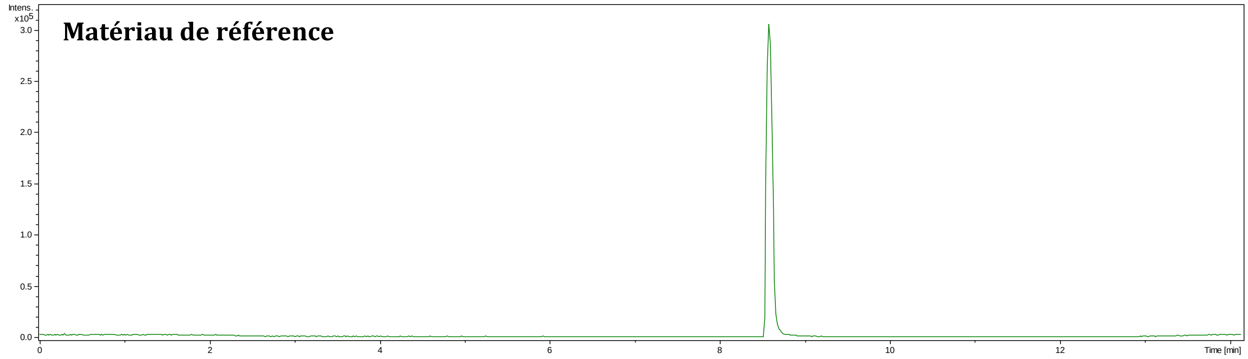

Figure 16: Masse extraite du matériau de référence (standards analytique CBD) (m/z 315.2315) détecté à 8.60 min en spectrométrie de masse à haute résolution (LC-Qtof)

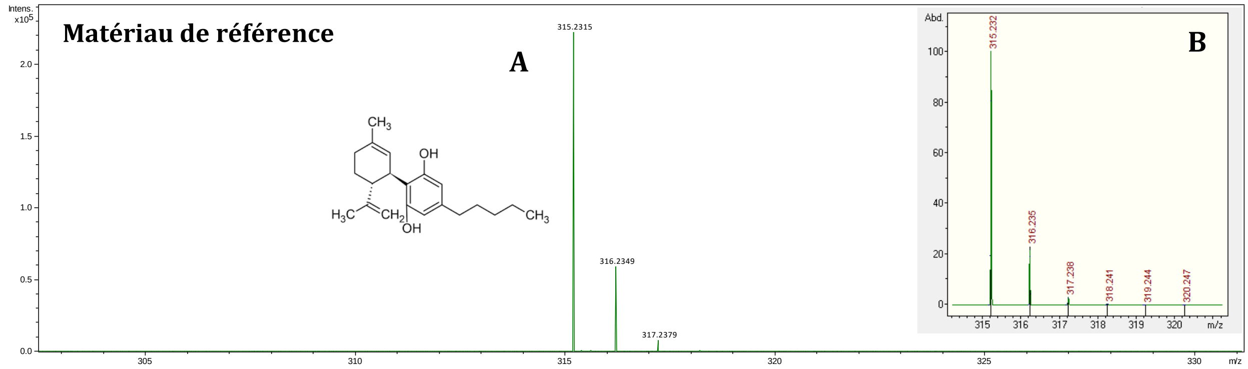

Figure 17: Confirmation du matériau de référence (standards analytique CBD) (m/z 315.2315) détecté à 8.60 min en spectrométrie de masse à haute résolution (LC-Qtof) avec le profil isotopique **(A)**, profil isotopique théorique du composé CBD **(B)**

FIN DU DOCUMENT
