# Supplementary material for: Buprenorphine and cannabidiol co-administration reduces survival in a mouse model of orthopedic trauma
Source: Front Pharmacol. 2025 Sep 11;16:1683842. doi: 10.3389/fphar.2025.1683842 (PMC12460340; doi:10.3389/fphar.2025.1683842)
Supplement: Supplementary file 2 [file Table1.docx]

*Supplementary Table 1. Disease Surveillance and Health Monitoring Criteria*

| **(A) Neurological Function** | **(B) Clinical Markers** |
| --- | --- |
| Righting reflex recovery time | Body weight |
| Post-anesthesia recovery time | Body temperature |
| Spontaneous locomotor activity | Mouse grimace scale |
|  | Posture |
|  | Coat condition |
|  | Nesting behavior |
|  | Limping |
|  | Wound site condition |
|  | Respiratory pattern |
|  | Abdominal bloating |
|  | Fecal output |

Mice were observed continuously for the first 8 hours post-injury, then every 6 hours during the first 24 hours, and subsequently three times daily. Monitoring was based on Neurological Function (A) and Clinical Markers (B).
