# Supplementary material for: Buprenorphine and cannabidiol co-administration reduces survival in a mouse model of orthopedic trauma
Source: Front Pharmacol. 2025 Sep 11;16:1683842. doi: 10.3389/fphar.2025.1683842 (PMC12460340; doi:10.3389/fphar.2025.1683842)
Supplement: Supplementary file 3 [file DataSheet1.pdf]

UNIVERSITÉ DE SHERBROOKE
Institut de pharmacologie de Sherbrooke (IPS)

Plateforme de bioanalyse
Campus de la santé,
3001 12 Ave N, Sherbrooke,
QC J1H 5N4
https://www.usherbrooke.ca/ips/fr/plateformes/bioanalyse/

Plateforme de Bioanalyse

Rapport d’étude

**Nom du requérant :**	Louis De Beaumont
**À l’attention de :**	Ian Massé
**Projet :**	Profil de pureté
**Date du rapport :**	2022-07-18
**Version :**	1
**Nombre de pages :**	7

Si vous désirez de l’information concernant cette analyse, S.V.P. contacter : bioanalysis-fmss@usherbrooke.ca

***Notes**

- 1 composé en poudre a été réceptionné : CBD
- Analyse en chromatographie liquide couplée à un détecteur à barrette de diode (LC-DAD)
- Analyse en chromatographie liquide couplée à un détecteur de masse (LC-Qtof)
- Le rapport contient une annexe : les profils du blanc procédural et du composé référence (standards analytiques du CBD) analysé par LC-DAD et LC-Qtof

Plateforme de bioanalyse
Campus de la santé,
3001 12 Ave N, Sherbrooke,
QC J1H 5N4
https://www.usherbrooke.ca/ips/fr/plateformes/bioanalyse/

Rapport d'analyse

1 Étude

**Critères**	**Paramètres**
Projet	Profil de pureté
Composé	CBD
Matrice	Poudre
Solvant de dissolution	Méthanol
Concentration	1 mg/mL
Pureté	Pureté déterminée par LC-DAD
Caractérisation	Caractérisation déterminée par LC-Qtof
Résultats	Profil UV et profil MS

2 Résultats

2.1 Profil de pureté du composé :

Composé	CBD
Structure	$C_{21}H_{30}O_2$
Analyse	LC-DAD
Temps de rétention	8.50 min
% pureté obtenue	≥97.64 %

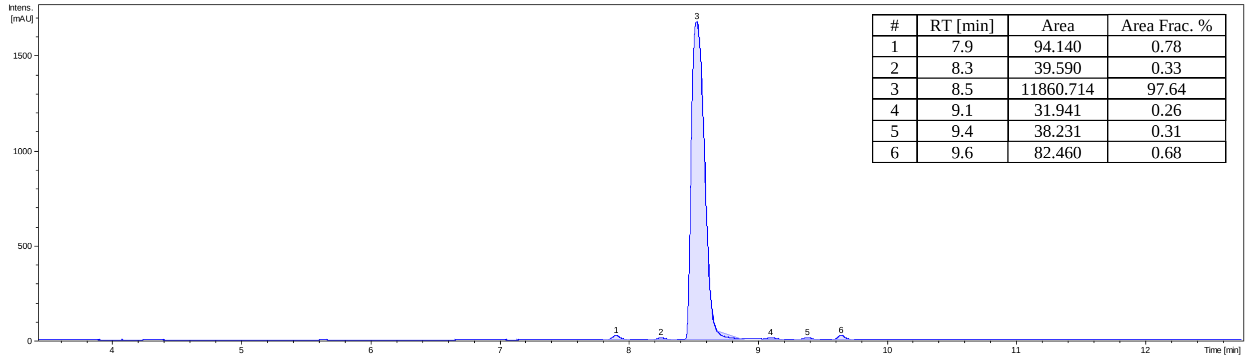

#	RT [min]	Area	Area Frac. %
1	7.9	94.140	0.78
2	8.3	39.590	0.33
3	8.5	11860.714	97.64
4	9.1	31.941	0.26
5	9.4	38.231	0.31
6	9.6	82.460	0.68

Figure 1: Profil de la solution de CBD obtenu en chromatographie liquide a barrette de diode (LC-DAD)

Un blanc procédural a été injecté et le profil en LC-DAD est présenté en annexe.

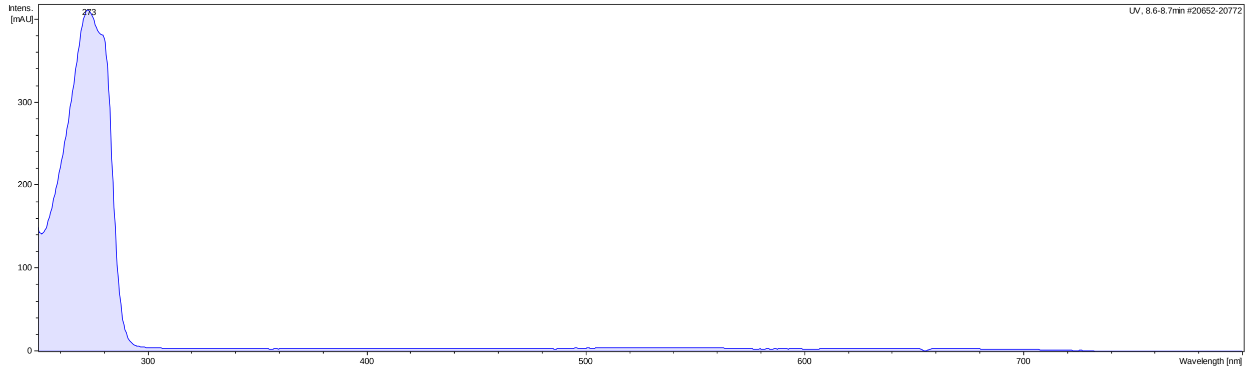

Figure 2: Spectre d'absorbance UV du composé majoritaire à 8.50 min

Un composé référence (standards analytique CBD) a été injecté afin de confirmer le profil UV en LC-DAD. Les profils sont présentés en annexe.

2.2 Profil de caractérisation du composé :

Composé	CBD
Structure	$C_{21}H_{30}O_2$
Analyse	LC-Qtof
Electrospray	ESI +
Temps de rétention	8.50 min
m/z théorique	315.2318
m/z mesuré	315.2321
Δm	0.0003

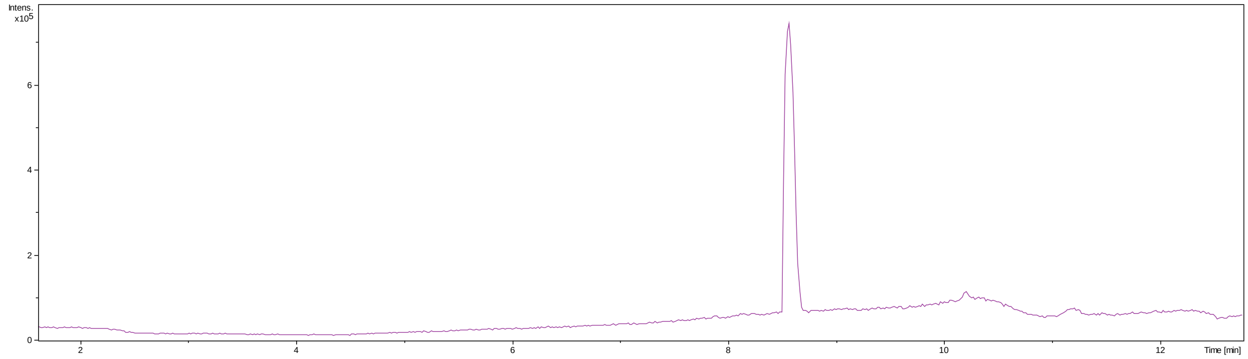

Figure 3: Profil chromatographique de la solution de CBD obtenu en spectrométrie de masse à haute résolution (LC-Qtof)

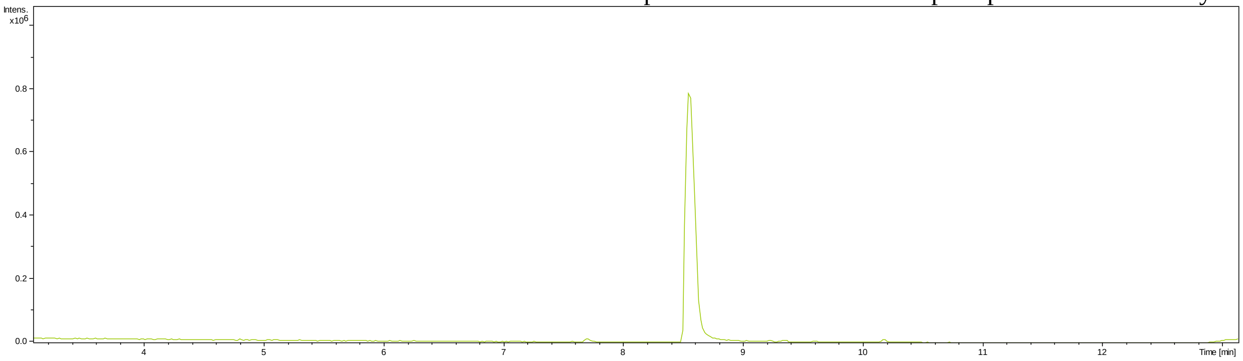

Figure 4: Masse extraite du composé majoritaire CBD (m/z 315.2321) détecté à 8.50 min en spectrométrie de masse à haute résolution (LC-Qtof)

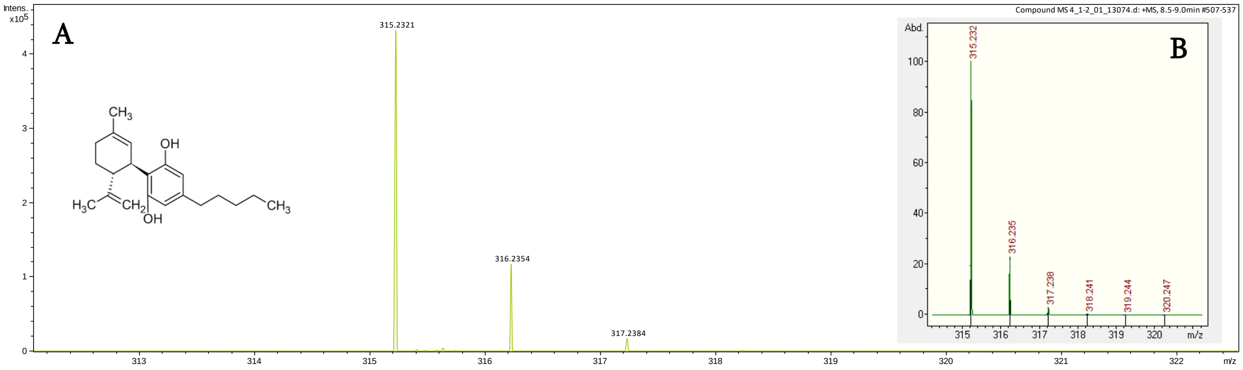

Figure 5: Confirmation du composé majoritaire CBD (m/z 315.2321) détecté à 8.50 min en spectrométrie de masse à haute résolution (LC-Qtof) avec le profil isotopique **(A)**, profil isotopique théorique du composé CBD **(B)**

La caractérisation et la confirmation du composé CBD a été réalisée par LC-Qtof et le profil isotopique du composé CBD est comparé au profil isotopique théorique.

Un blanc procédural a été injecté et le profil en LC-Qtof est présenté en annexe.

Un composé référence (standards analytique CBD) a été injecté afin de confirmer la caractérisation du composé CBD par LC-Qtof. Les profils sont représentés en annexe.

3 Bilan

- 1 composé en poudre a été réceptionné : le composé a été mis en solution dans du méthanol à une concentration de 1mg/mL
- Le composé CBD montre une pureté > 97% en LC-DAD
- Le Pic majoritaire à 8.50 min a été identifié et confirmé comme étant du CBD par LC-Qtof
- L'analyse d'un composé référence (standards analytique CBD) a permis de confirmer et caractériser le composé CBD réceptionné.
- Présence d'impureté en LC-DAD et non identifiables en LC-Qtof

4 Annexe :

Cette section annexe regroupe les profils en LC-DAD et LC-Qtof du blanc procédural et du composé référence (standards analytique CBD).

4.1 Profil du blanc procédural

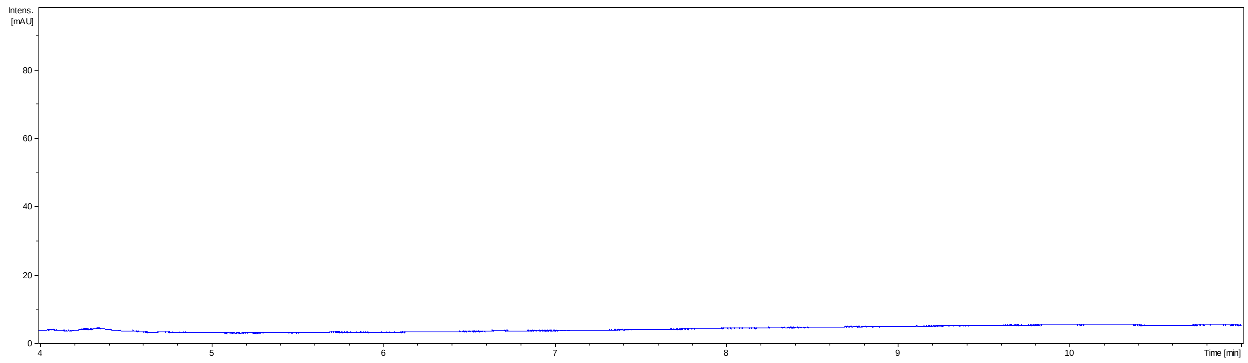

Figure 6: Profil du blanc procédural en chromatographie liquide a barrette de diode (LC-DAD)

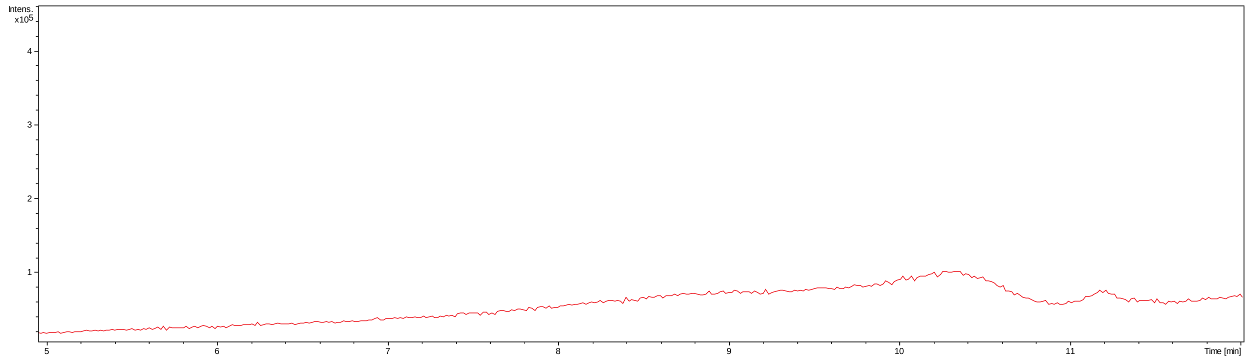

Figure 7: Profil du blanc procédural en spectrométrie de masse à haute résolution (LC-Qtof)

4.2 Profil du composé référence (standards analytique CBD)

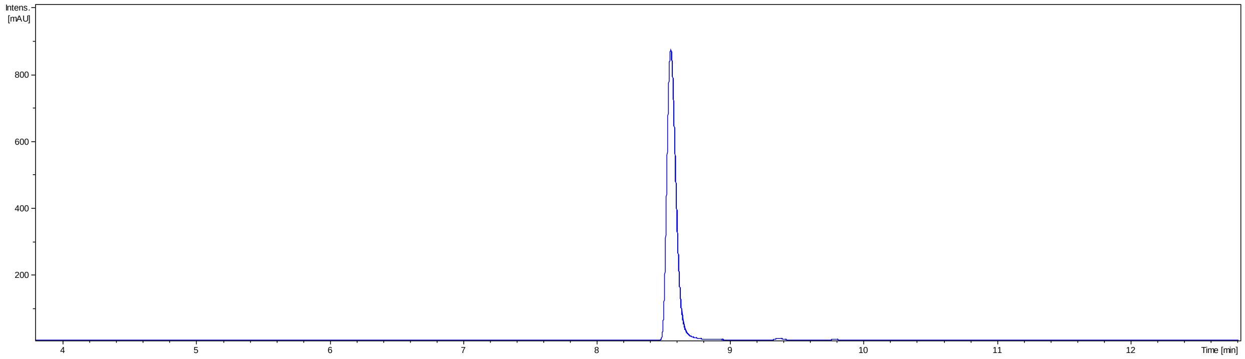

Figure 8: Profil du composé référence (standards analytique) obtenu en chromatographie liquide a barrette de diode (LC-DAD)

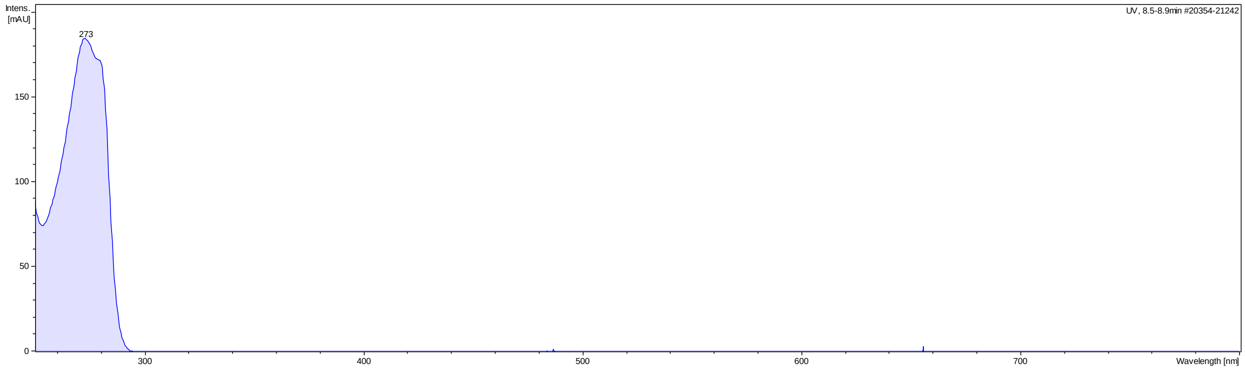

Figure 9: Spectre d'absorbance UV du composé référence (standards analytique) à 8.50 min

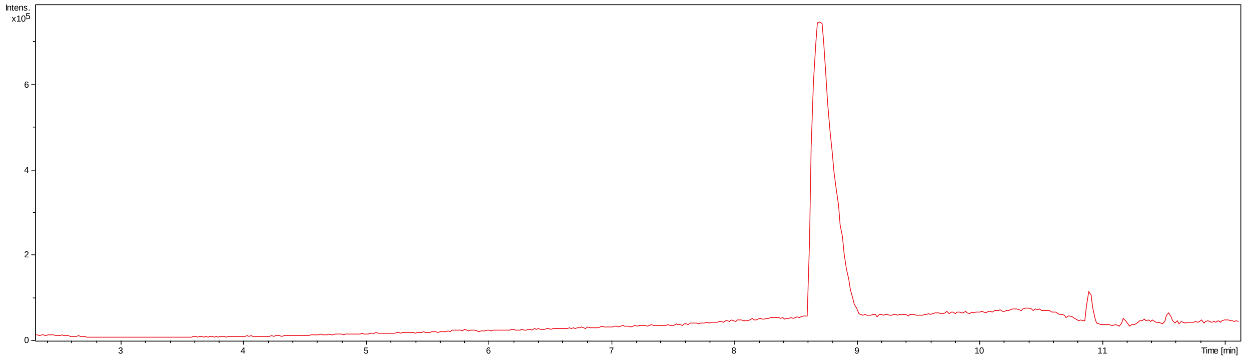

Figure 10: Profil chromatographique du composé référence (standards analytique CBD) obtenu en spectrométrie de masse à haute résolution (LC-Qtof)

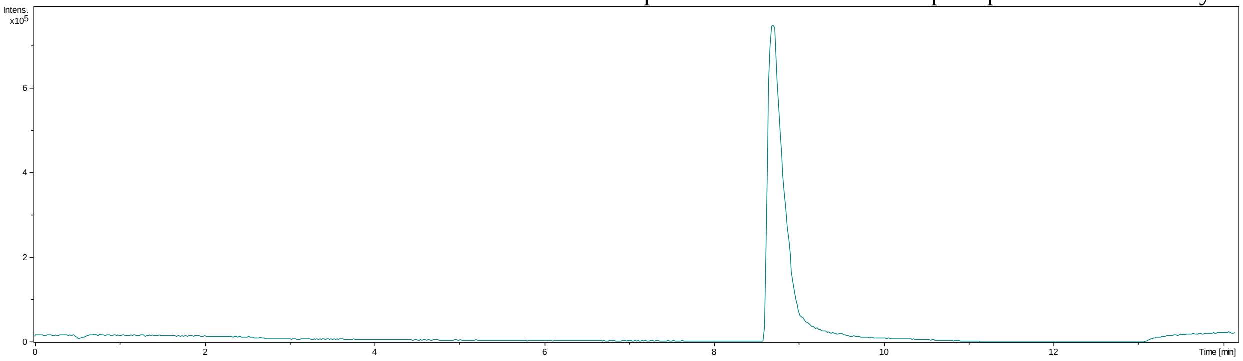

Figure 11: Masse extraite du composé référence (standards analytique) CBD (m/z 315.2326) détecté à 8.50 min en spectrométrie de masse à haute résolution (LC-Qtof)

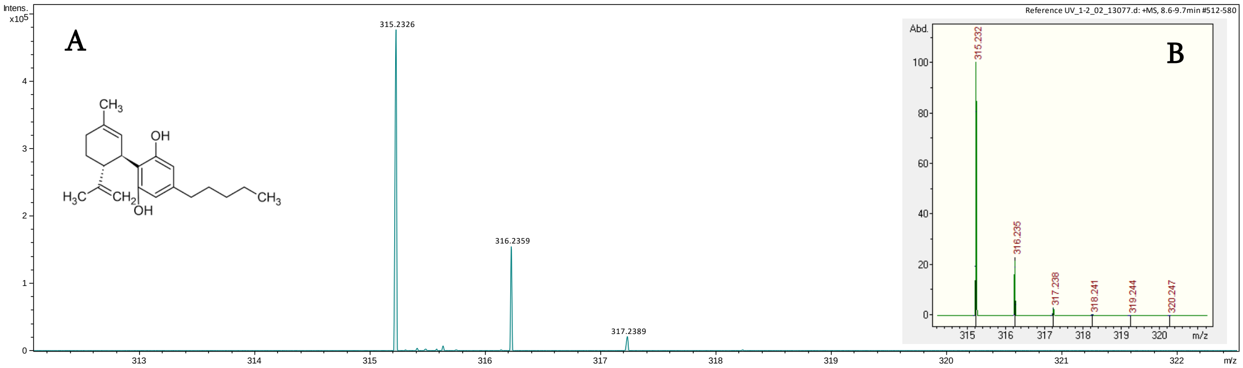

Figure 12: Confirmation du composé référence (standards analytique) CBD (m/z 315.2326) détecté à 8.50 min en spectrométrie de masse à haute résolution (LC-Qtof) avec le profil isotopique **(A)**, profil isotopique théorique du composé CBD **(B)**

FIN DU DOCUMENT
